
JOURNAL INFORMATION
==============================
NLM Title Abbreviation: Exp Physiol
Journal ID: Exp Physiol
NLM Journal ID: 9002940
Journal ID: EPH

Experimental Physiology

ISSN: 0958-0670
EISSN: 1469-445X
Publisher: Wiley

ARTICLE INFORMATION
==============================
PMCID: PMC13238884
DOI: 10.1113/eph.70327
Article ID: EPH70327
Article version: 1
Subjects: Issue Information, Issue Information

Issue Information

Electronic publication date: 2026 Jun 1
Publication date: 2026 Jun
Volume: 111
Issue: 6
Issue title: Special Issue: Exercise as Medicine
First page: 3101
Last page: 3105
Copyright: © 2026 The Authors. Experimental Physiology published by John Wiley & Sons Ltd on behalf of The Physiological Society.
License: This is an open access article under the terms of the http://creativecommons.org/licenses/by/4.0/ License, which permits use, distribution and reproduction in any medium, provided the original work is properly cited.
License URL: https://creativecommons.org/licenses/by/4.0/

Figure count: 0
Table count: 0
Page count: 5

source-schema-version-number: 2.0
cover-date: 1 June 2026
details-of-publishers-convertor: Converter:WILEY_ML3GV2_TO_JATSPMC version:6.8.3 mode:remove_FC converted:09.07.2026

==============================

### Experimental Physiology

Open Access

### Exercise as Medicine

Volume 111 | Issue 6 | 1 June 2026

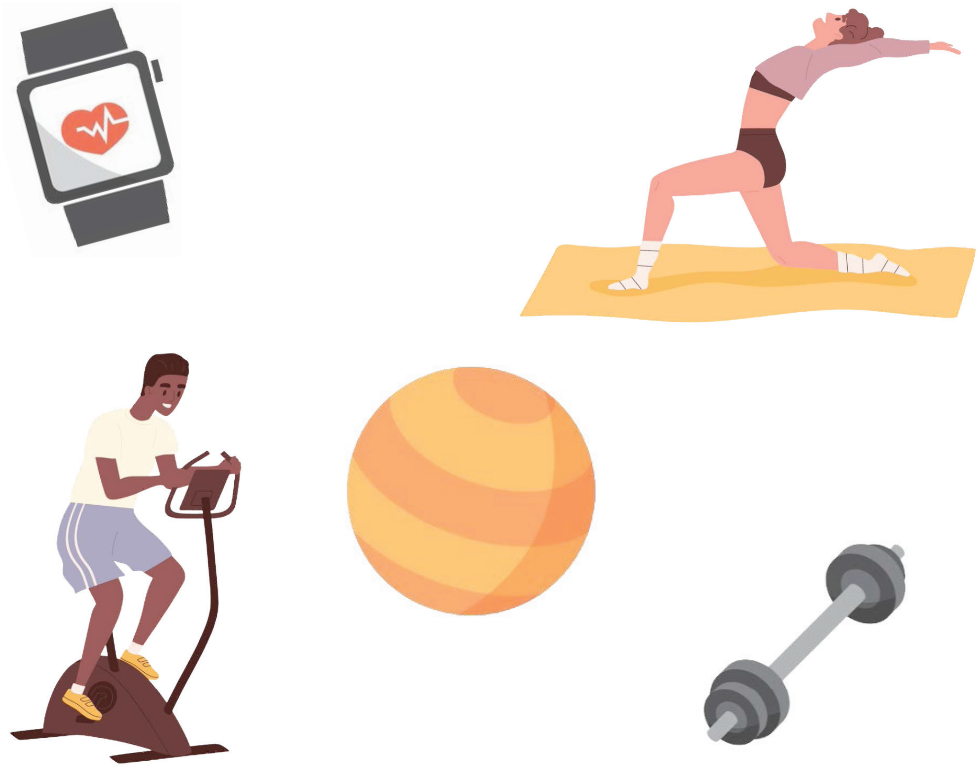

WILEY

The Physiological Society

150 YEARS 1876 2026 LEADING DISCOVERY SCIENCE

Experimental Physiology
Open Access
Integration and Translation

Volume 111/Issue 6/
1 June 2026

### EDITORIAL BOARD

**EDITOR-IN-CHIEF**
Damian Bailey *Pontypridd, UK*

**DEPUTY EDITOR-IN-CHIEF EUROPE**
Ronan Berg *Copenhagen, Denmark*

**DEPUTY EDITOR-IN-CHIEF USA**
David Poole *Manhattan, KS, USA*

**SENIOR ETHICS EDITOR**
Susan Barman *East Lansing, MI, USA*

**STATISTICAL EDITOR**
Cody Durrer *Copenhagen, Denmark*

**SENIOR REVIEWS EDITOR**
Toby Mündel *Ontario, Canada*

**CONNECTIONS EDITOR**
Prem Kumar *Birmingham, UK*

**SENIOR EDITORS**
Dana Almohazey *Dammam, Saudi Arabia*
Jill Barnes *Madison, WI, USA*
R. Matthew Brothers *Arlington, TX, USA*
Kanokwan 'Mandy' Bunsawat *Iowa City, IA, USA*
Kirstene Callø *Copenhagen, Denmark*
Katrina Colafella *Victoria, Australia*
Ellen Dawson *Liverpool, UK*
Colleen Deane *Southampton, UK*
Peter Zu Eulenburg *Munich, Germany*
Federico Formenti *London, UK*
Julien Ochala *Copenhagen, Denmark*
Shigehiko Ogoh *Kawagoe, Japan*
Jason Peart *Gold Coast, Australia*
Peter Rasmussen *Copenhagen, Denmark*
Lee Romer *London, UK*
Harry B. Rossiter *Los Angeles, CA, USA*
Bill Sheel *Vancouver, Canada*
Mike Stembridge *Cardiff, UK*

**REVIEWING EDITORS**
Tatsuro Amano *Niigata, Japan*
Claire Badenhorst *Palmerston North, NZ*
Tracy Baynard *Boston, MA, USA*
Hannah Caldwell *Copenhagen, Denmark*
Havovi Chichger *Cambridge, UK*
Silvia Conde *Lisbon, Portugal*
Joseph Costello *Portsmouth, UK*
Jennifer Davies *Cardiff, UK*
Judy Delp *Tallahassee, FL, USA*
Aimee Drane *Cardiff, UK*
Esther Dupont-Versteegden *Lexington, KY, USA*
Andrea Fellet *Buenos Aires, Argentina*
Carrie Ferguson *Torrance, CA, USA*
Igor Fernandes *Niteroi, Brazil*
Ryan Hoiland *Kelowna, Canada*
Seth Holwerda *Kansas City, KS, USA*
Anna Hudson *Adelaide, Australia*
Matthew Kay *Washington, DC, USA*
James Lang *Ames, Iowa, USA*
Jacqueline Limberg *Columbia, MO, USA*
Fiona McBryde *Auckland, NZ*
Carmen De Miguel *Birmingham, AL, USA*
Niels Olesen *Copenhagen, Denmark*
Leah Reznikov *Gainesville, FL, USA*
Kleiton Silva *Camden, NJ, USA*
Sarah Short *Iowa City, IA, USA*
Nathaniel Szewczyk *Nottingham, UK*
Can Ozan Tan *Boston, MA, USA*
Mike Tymko *Vancouver, Canada*
Kai Yang *Guangzhou, China*
Song Yao *Melbourne, Australia*
Gensheng Zhang *Hangzhou, China*

**FUTURE LEADERS**
Edward Ashworth *Basel, Switzerland*
Sophie Broome *Melbourne, Australia*
Callum Brownstein *Newcastle, UK*
Daniel Craighead *Minneapolis, MN, USA*
Robert Lakin *Toronto, Canada*
Jedd Pratt *Manchester, UK*
Fabio Sarto *Padua, Italy*
Hollie Speer *Canberra, Australia*

**CONSULTANT EDITORS**
Richard Burden *Bisham, UK*
Nic Green *Bedfordshire, UK*
Julie Greeves *Salisbury, UK*
Denis Noble *Oxford, UK*
Nanduri Prabhakar *Chicago, IL, USA*

**PUBLISHER**
Alex Stewart (astewart@physoc.org)

**DEPUTY MANAGING EDITOR**
Hollie Evans (hevans@physoc.org)

**PEER REVIEW MANAGER**
Diana Jones (ephjournal@physoc.org)

**PEER REVIEW COORDINATOR**
Paroma Guha (ephjournal@physoc.org)

**EDITORIAL OFFICE**
The Physiological Society
Hodgkin Huxley House
30 Farringdon Lane, Clerkenwell
London EC1R 3AW

**PRODUCTION OFFICE**
K. Nandini (EPH@wiley.com)

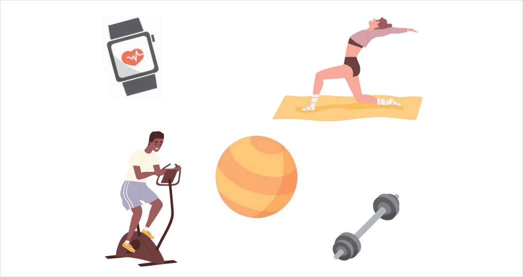

**Cover Illustration**
A representation of the Exercise as Medicine special issue. This special issue was organised Professor Ronan Berg (University of Copenhagen), Dr Colleen Deane (University of Southampton), Dr Jill Barnes (University of Wisconsin-Madison) and Professor Harry Rossiter (The Lundquist Institute for Biomedical Innovation at Harbor-UCLA Medical Center).

**Open Access and Copyright**
All articles published by Experimental Physiology [submitted after 18 October 2022] are fully open access: immediately freely available to read, download, and share. All articles [accepted from 18 October 2022] are published under the terms of a Creative Commons license.
Copyright on any research article published by Experimental Physiology is retained by the author(s).
Further information about open access licenses and copyright can be found at: https://authorservices.wiley.com/author-resources/Journal-Authors/licensing/licensing-info-faqs.html#4. Information on the licenses used by Experimental Physiology can be found at: https://physoc.onlinelibrary.wiley.com/journal/1469445X.

For submission instructions and all other information visit: https://physoc.onlinelibrary.wiley.com/journal/1469445X

The Physiological Society and The Physiological Society logo are trade marks belonging to The Physiological Society and are registered in the UK and in the EU Community, respectively.

*Experimental Physiology* publishes high quality, original, physiological research papers that give novel insights into fundamental homeostatic and adaptive responses in health and papers that further our knowledge of pathophysiological mechanisms in diseases.

Experimental Physiology
Open Access
Integration and Translation

### INFORMATION AND GUIDANCE FOR AUTHORS

**Full version online at http://ep.msubmit.net/cgi-bin/main.plex?form_type=display_requirements**

### ORIGIN

*The Quarterly Journal of Experimental Physiology*, founded in 1908 by Sir Edward Sharpey-Schafer, Professor of Physiology, University of Edinburgh, became a publication of The Physiological Society in 1981. The title was changed to *Experimental Physiology* in 1990.

### SCOPE

*Experimental Physiology* publishes research papers that report novel insights into homeostatic and adaptive responses in health, as well as those that further our understanding of pathophysiological mechanisms in disease. We encourage papers that embrace the journal's orientation of translation and integration, including studies of the adaptive responses to exercise, acute and chronic environmental stressors, growth and aging, and diseases where integrative homeostatic mechanisms play a key role in the response to and evolution of the disease process. Examples of such diseases include hypertension, heart failure, hypoxic lung disease, endocrine and neurological disorders. We are also keen to publish research that has a translational aspect or clinical application. Comparative physiology work that can be applied to aid the understanding human physiology is also encouraged.

Manuscripts that report the use of bioinformatic, genomic, molecular, proteomic and cellular techniques to provide novel insights into integrative physiological and pathophysiological mechanisms are welcomed.

We are also interested in methodological papers reporting important new developments in physiological techniques that could lead to significant new insights.

Papers which observe a significant new physiological behaviour or phenomenon which has the potential to alter the understanding of that field, but which have not yet identified the mechanisms may also be considered. Such papers should demonstrate the experimental examination and exclusion of a number of plausible mechanisms for the important novel phenomenon.

### Article Types

In addition to original research, *Experimental Physiology* consider the following article types for publication:

*Case studies, Short Communication, Connections, Reviews, Full Reviews, Hot Topic Reviews Myths and Methodologies and Symposium Reports.*

Review articles are normally by invitation but proposals may be submitted for consideration.

Commentary articles in the form of Viewpoints and Letters are also published.

For full guidelines on all published article see full guidelines.

C O P E COMMITTEE ON PUBLICATION ETHICS

This journal is a member of and subscribes to the principles of the Committee on Publication Ethics

### ETHICS OF EXPERIMENTATION

**Animal Experiments** - see full journal policy at https://physoc.onlinelibrary.wiley.com/hub/animal-experiments

Experimental Physiology endorses the ARRIVE Guidelines for reporting *in vivo* experiments: http://onlinelibrary.wiley.com/doi/10.1113/expphysiol.2010.053793/full

Authors of papers submitted to Experimental Physiology must adhere to the journals' principles and standards for reporting animal experiments.

Authors should read these guidelines to ensure that their research and experiments adhere to our exacting standards.

Euthanasia/humane killing: Animals must be killed using methods approved for that species, stage of development and size. The standards required by the journals are those of the UK/European Union legislation, set out in in ASPA Schedule 1 in the UK and in Annex IV in the European Directive 2010/63/EU. Methods not on Schedule 1 or Annex IV could be permissible but even where the method used conforms with national guidelines, the onus is on the author to provide scientific evidence to demonstrate that it is at least as humane as those on Schedule 1 and Annex IV.

Our guidelines have been outlined in the Editorial 'Principles and standards for reporting animal experiments in Experimental Physiology'.

**Confirmation of compliance**

Authors must state in the Methods section that the investigators understand the ethical principles under which the journal operates and that their work complies with the animal ethics checklist as outlined in the Editorial.

**Human Experiments** - See full journal policy at https://physoc.onlinelibrary.wiley.com/hub/human-experiments

The Editorial Boards are concerned that all work published in The Journal of Physiology and Experimental Physiology should have been conducted according to the highest standards of safety and ethics. Legislation and accepted practice concerning human experimentation vary from country to country and consequently it is difficult to define absolute requirements. However, work with human subjects should conform to the standards set by the Declaration of Helsinki (last modified in 2013), the Medical Research Council's online guidance on the use of human tissue, and the guidelines set out below. The objectives must be to ensure that all risks are minimized and that subjects are not injured and do not feel they have been abused as a result of participating in the study. Any definition of abuse will include excessive or unexpected pain or discomfort experienced during the experiment. Note, in the case of experiments involving minors, any risks must have been considered absent or minimal, and evidence must be presented that the experiments were performed with the understanding and consent of the legal guardian.

All manuscripts must contain statements indicating that (i) informed consent has been obtained, preferably in writing, (ii) studies conformed to the standards set by the Declaration of Helsinki or the version that was in place at the time of the experiments, NOTE: If the research study was registered (clause 35 of the Declaration of Helsinki) the registration database should be indicated, else the lack of registration should be noted as an exception (e.g. The study conformed to the standards set by the Declaration of Helsinki, except for registration in a database.); and (iii) the procedures have been approved by the local ethics committee.

### DISCLAIMER

The Publisher, the Physiological Society and Editors cannot be held responsible for any errors in or any consequences arising from the use of information contained in this journal. The views and opinions expressed do not necessarily reflect those of the Publisher, the Physiological Society or Editors, neither does the publication of advertisements constitute any endorsement by the Publisher, the Physiological Society, Editors, or Authors of the products advertised.

Wiley Open Access articles posted to repositories or websites are without warranty from Wiley of any kind, either express or implied, including, but not limited to, warranties of merchantability, fitness for a particular purpose, or non-infringement. To the fullest extent permitted by law Wiley disclaims all liability for any loss or damage arising out of, or in connection with, the use of or inability to use the content.

### Experimental Physiology

Open Access

### Exercise as Medicine

Volume 111 | Issue 6 | 1 June 2026

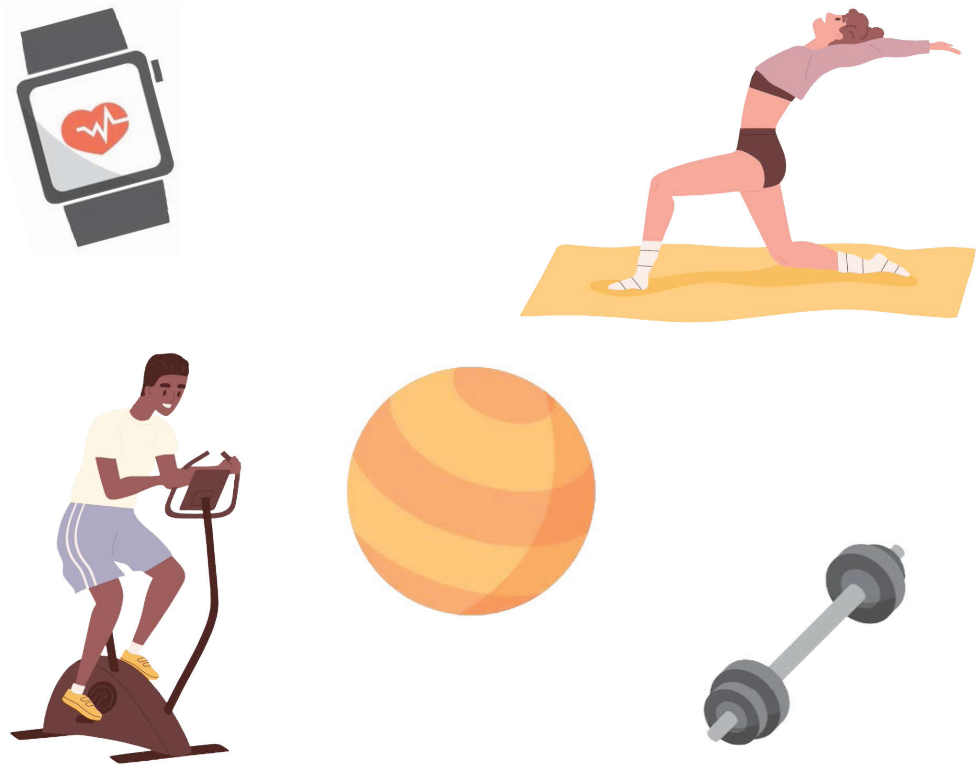

WILEY

The Physiological Society

150 YEARS 1876 2026 LEADING DISCOVERY SCIENCE

Experimental Physiology
Open Access
Integration and Translation

Volume 111/Issue 6/
1 June 2026

### EDITORIAL BOARD

**EDITOR-IN-CHIEF**
Damian Bailey *Pontypridd, UK*

**DEPUTY EDITOR-IN-CHIEF EUROPE**
Ronan Berg *Copenhagen, Denmark*

**DEPUTY EDITOR-IN-CHIEF USA**
David Poole *Manhattan, KS, USA*

**SENIOR ETHICS EDITOR**
Susan Barman *East Lansing, MI, USA*

**STATISTICAL EDITOR**
Cody Durrer *Copenhagen, Denmark*

**SENIOR REVIEWS EDITOR**
Toby Mündel *Ontario, Canada*

**CONNECTIONS EDITOR**
Prem Kumar *Birmingham, UK*

**SENIOR EDITORS**
Dana Almohazey *Dammam, Saudi Arabia*
Jill Barnes *Madison, WI, USA*
R. Matthew Brothers *Arlington, TX, USA*
Kanokwan 'Mandy' Bunsawat *Iowa City, IA, USA*
Kirstene Callø *Copenhagen, Denmark*
Katrina Colafella *Victoria, Australia*
Ellen Dawson *Liverpool, UK*
Colleen Deane *Southampton, UK*
Peter Zu Eulenburg *Munich, Germany*
Federico Formenti *London, UK*
Julien Ochala *Copenhagen, Denmark*
Shigehiko Ogoh *Kawagoe, Japan*
Jason Peart *Gold Coast, Australia*
Peter Rasmussen *Copenhagen, Denmark*
Lee Romer *London, UK*
Harry B. Rossiter *Los Angeles, CA, USA*
Bill Sheel *Vancouver, Canada*
Mike Stembridge *Cardiff, UK*

**REVIEWING EDITORS**
Tatsuro Amano *Niigata, Japan*
Claire Badenhorst *Palmerston North, NZ*
Tracy Baynard *Boston, MA, USA*
Hannah Caldwell *Copenhagen, Denmark*
Havovi Chichger *Cambridge, UK*
Silvia Conde *Lisbon, Portugal*
Joseph Costello *Portsmouth, UK*
Jennifer Davies *Cardiff, UK*
Judy Delp *Tallahassee, FL, USA*
Aimee Drane *Cardiff, UK*
Esther Dupont-Versteegden *Lexington, KY, USA*
Andrea Fellet *Buenos Aires, Argentina*
Carrie Ferguson *Torrance, CA, USA*
Igor Fernandes *Niteroi, Brazil*
Ryan Hoiland *Kelowna, Canada*
Seth Holwerda *Kansas City, KS, USA*
Anna Hudson *Adelaide, Australia*
Matthew Kay *Washington, DC, USA*
James Lang *Ames, Iowa, USA*
Jacqueline Limberg *Columbia, MO, USA*
Fiona McBryde *Auckland, NZ*
Carmen De Miguel *Birmingham, AL, USA*
Niels Olesen *Copenhagen, Denmark*
Leah Reznikov *Gainesville, FL, USA*
Kleiton Silva *Camden, NJ, USA*
Sarah Short *Iowa City, IA, USA*
Nathaniel Szewczyk *Nottingham, UK*
Can Ozan Tan *Boston, MA, USA*
Mike Tymko *Vancouver, Canada*
Kai Yang *Guangzhou, China*
Song Yao *Melbourne, Australia*
Gensheng Zhang *Hangzhou, China*

**FUTURE LEADERS**
Edward Ashworth *Basel, Switzerland*
Sophie Broome *Melbourne, Australia*
Callum Brownstein *Newcastle, UK*
Daniel Craighead *Minneapolis, MN, USA*
Robert Lakin *Toronto, Canada*
Jedd Pratt *Manchester, UK*
Fabio Sarto *Padua, Italy*
Hollie Speer *Canberra, Australia*

**CONSULTANT EDITORS**
Richard Burden *Bisham, UK*
Nic Green *Bedfordshire, UK*
Julie Greeves *Salisbury, UK*
Denis Noble *Oxford, UK*
Nanduri Prabhakar *Chicago, IL, USA*

**PUBLISHER**
Alex Stewart (astewart@physoc.org)

**DEPUTY MANAGING EDITOR**
Hollie Evans (hevans@physoc.org)

**PEER REVIEW MANAGER**
Diana Jones (ephjournal@physoc.org)

**PEER REVIEW COORDINATOR**
Paroma Guha (ephjournal@physoc.org)

**EDITORIAL OFFICE**
The Physiological Society
Hodgkin Huxley House
30 Farringdon Lane, Clerkenwell
London EC1R 3AW

**PRODUCTION OFFICE**
K. Nandini (EPH@wiley.com)

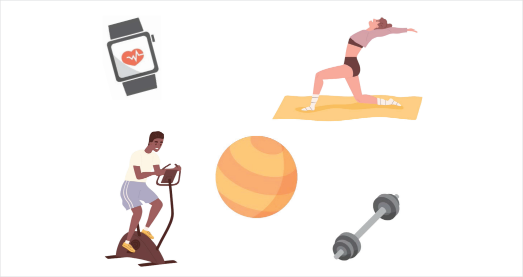

**Cover Illustration**
A representation of the Exercise as Medicine special issue. This special issue was organised Professor Ronan Berg (University of Copenhagen), Dr Colleen Deane (University of Southampton), Dr Jill Barnes (University of Wisconsin-Madison) and Professor Harry Rossiter (The Lundquist Institute for Biomedical Innovation at Harbor-UCLA Medical Center).

**Open Access and Copyright**
All articles published by Experimental Physiology [submitted after 18 October 2022] are fully open access: immediately freely available to read, download, and share. All articles [accepted from 18 October 2022] are published under the terms of a Creative Commons license.
Copyright on any research article published by Experimental Physiology is retained by the author(s).
Further information about open access licenses and copyright can be found at: https://authorservices.wiley.com/author-resources/Journal-Authors/licensing/licensing-info-faqs.html#4. Information on the licenses used by Experimental Physiology can be found at: https://physoc.onlinelibrary.wiley.com/journal/1469445X.

For submission instructions and all other information visit: https://physoc.onlinelibrary.wiley.com/journal/1469445X

The Physiological Society and The Physiological Society logo are trade marks belonging to The Physiological Society and are registered in the UK and in the EU Community, respectively.

*Experimental Physiology* publishes high quality, original, physiological research papers that give novel insights into fundamental homeostatic and adaptive responses in health and papers that further our knowledge of pathophysiological mechanisms in diseases.

Experimental Physiology
Open Access
Integration and Translation

### INFORMATION AND GUIDANCE FOR AUTHORS

### Full version online at http://ep.msubmit.net/cgi-bin/main.plex?form_type=display_requirements

### ORIGIN

*The Quarterly Journal of Experimental Physiology*, founded in 1908 by Sir Edward Sharpey-Schafer, Professor of Physiology, University of Edinburgh, became a publication of The Physiological Society in 1981. The title was changed to *Experimental Physiology* in 1990.

### SCOPE

*Experimental Physiology* publishes research papers that report novel insights into homeostatic and adaptive responses in health, as well as those that further our understanding of pathophysiological mechanisms in disease. We encourage papers that embrace the journal's orientation of translation and integration, including studies of the adaptive responses to exercise, acute and chronic environmental stressors, growth and aging, and diseases where integrative homeostatic mechanisms play a key role in the response to and evolution of the disease process. Examples of such diseases include hypertension, heart failure, hypoxic lung disease, endocrine and neurological disorders. We are also keen to publish research that has a translational aspect or clinical application. Comparative physiology work that can be applied to aid the understanding human physiology is also encouraged.

Manuscripts that report the use of bioinformatic, genomic, molecular, proteomic and cellular techniques to provide novel insights into integrative physiological and pathophysiological mechanisms are welcomed.

We are also interested in methodological papers reporting important new developments in physiological techniques that could lead to significant new insights.

Papers which observe a significant new physiological behaviour or phenomenon which has the potential to alter the understanding of that field, but which have not yet identified the mechanisms may also be considered. Such papers should demonstrate the experimental examination and exclusion of a number of plausible mechanisms for the important novel phenomenon.

### Article Types

In addition to original research, *Experimental Physiology* consider the following article types for publication:

*Case studies, Short Communication, Connections, Reviews, Full Reviews, Hot Topic Reviews Myths and Methodologies and Symposium Reports.*

Review articles are normally by invitation but proposals may be submitted for consideration.

Commentary articles in the form of Viewpoints and Letters are also published.

For full guidelines on all published article see full guidelines.

C O P E COMMITTEE ON PUBLICATION ETHICS

This journal is a member of and subscribes to the principles of the Committee on Publication Ethics

### ETHICS OF EXPERIMENTATION

**Animal Experiments** - see full journal policy at https://physoc.onlinelibrary.wiley.com/hub/animal-experiments

Experimental Physiology endorses the ARRIVE Guidelines for reporting *in vivo* experiments: http://onlinelibrary.wiley.com/doi/10.1113/expphysiol.2010.053793/full

Authors of papers submitted to Experimental Physiology must adhere to the journals' principles and standards for reporting animal experiments.

Authors should read these guidelines to ensure that their research and experiments adhere to our exacting standards.

Euthanasia/humane killing: Animals must be killed using methods approved for that species, stage of development and size. The standards required by the journals are those of the UK/European Union legislation, set out in in ASPA Schedule 1 in the UK and in Annex IV in the European Directive 2010/63/EU. Methods not on Schedule 1 or Annex IV could be permissible but even where the method used conforms with national guidelines, the onus is on the author to provide scientific evidence to demonstrate that it is at least as humane as those on Schedule 1 and Annex IV.

Our guidelines have been outlined in the Editorial 'Principles and standards for reporting animal experiments in Experimental Physiology'.

**Confirmation of compliance**

Authors must state in the Methods section that the investigators understand the ethical principles under which the journal operates and that their work complies with the animal ethics checklist as outlined in the Editorial.

**Human Experiments** - See full journal policy at https://physoc.onlinelibrary.wiley.com/hub/human-experiments

The Editorial Boards are concerned that all work published in The Journal of Physiology and Experimental Physiology should have been conducted according to the highest standards of safety and ethics. Legislation and accepted practice concerning human experimentation vary from country to country and consequently it is difficult to define absolute requirements. However, work with human subjects should conform to the standards set by the Declaration of Helsinki (last modified in 2013), the Medical Research Council's online guidance on the use of human tissue, and the guidelines set out below. The objectives must be to ensure that all risks are minimized and that subjects are not injured and do not feel they have been abused as a result of participating in the study. Any definition of abuse will include excessive or unexpected pain or discomfort experienced during the experiment. Note, in the case of experiments involving minors, any risks must have been considered absent or minimal, and evidence must be presented that the experiments were performed with the understanding and consent of the legal guardian.

All manuscripts must contain statements indicating that (i) informed consent has been obtained, preferably in writing, (ii) studies conformed to the standards set by the Declaration of Helsinki or the version that was in place at the time of the experiments, NOTE: If the research study was registered (clause 35 of the Declaration of Helsinki) the registration database should be indicated, else the lack of registration should be noted as an exception (e.g. The study conformed to the standards set by the Declaration of Helsinki, except for registration in a database.); and (iii) the procedures have been approved by the local ethics committee.

### DISCLAIMER

The Publisher, the Physiological Society and Editors cannot be held responsible for any errors in or any consequences arising from the use of information contained in this journal. The views and opinions expressed do not necessarily reflect those of the Publisher, the Physiological Society or Editors, neither does the publication of advertisements constitute any endorsement by the Publisher, the Physiological Society, Editors, or Authors of the products advertised.

Wiley Open Access articles posted to repositories or websites are without warranty from Wiley of any kind, either express or implied, including, but not limited to, warranties of merchantability, fitness for a particular purpose, or non-infringement. To the fullest extent permitted by law Wiley disclaims all liability for any loss or damage arising out of, or in connection with, the use of or inability to use the content.

### Experimental Physiology

Open Access

### Exercise as Medicine

Volume 111 | Issue 6 | 1 June 2026

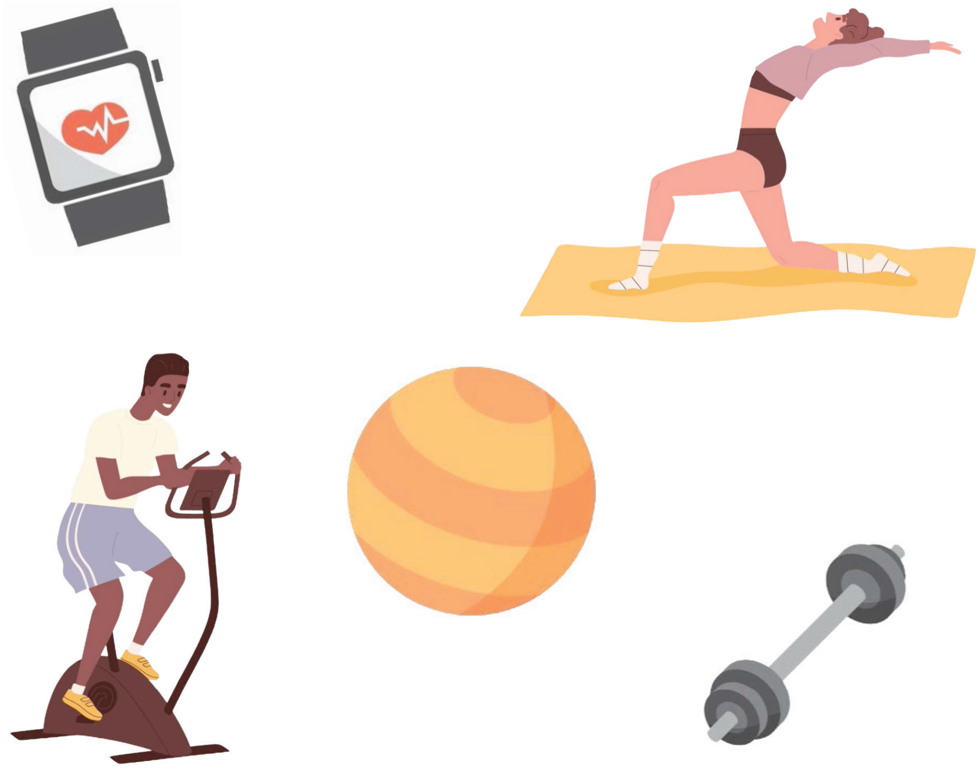

WILEY

The Physiological Society

150 YEARS 1876 2026 LEADING DISCOVERY SCIENCE

Experimental Physiology
Open Access
Integration and Translation

Volume 111/Issue 6/
1 June 2026

### EDITORIAL BOARD

**EDITOR-IN-CHIEF**
Damian Bailey *Pontypridd, UK*

**DEPUTY EDITOR-IN-CHIEF EUROPE**
Ronan Berg *Copenhagen, Denmark*

**DEPUTY EDITOR-IN-CHIEF USA**
David Poole *Manhattan, KS, USA*

**SENIOR ETHICS EDITOR**
Susan Barman *East Lansing, MI, USA*

**STATISTICAL EDITOR**
Cody Durrer *Copenhagen, Denmark*

**SENIOR REVIEWS EDITOR**
Toby Mündel *Ontario, Canada*

**CONNECTIONS EDITOR**
Prem Kumar *Birmingham, UK*

**SENIOR EDITORS**
Dana Almohazey *Dammam, Saudi Arabia*
Jill Barnes *Madison, WI, USA*
R. Matthew Brothers *Arlington, TX, USA*
Kanokwan 'Mandy' Bunsawat *Iowa City, IA, USA*
Kirstene Callø *Copenhagen, Denmark*
Katrina Colafella *Victoria, Australia*
Ellen Dawson *Liverpool, UK*
Colleen Deane *Southampton, UK*
Peter Zu Eulenburg *Munich, Germany*
Federico Formenti *London, UK*
Julien Ochala *Copenhagen, Denmark*
Shigehiko Ogoh *Kawagoe, Japan*
Jason Peart *Gold Coast, Australia*
Peter Rasmussen *Copenhagen, Denmark*
Lee Romer *London, UK*
Harry B. Rossiter *Los Angeles, CA, USA*
Bill Sheel *Vancouver, Canada*
Mike Stembridge *Cardiff, UK*

**REVIEWING EDITORS**
Tatsuro Amano *Niigata, Japan*
Claire Badenhorst *Palmerston North, NZ*
Tracy Baynard *Boston, MA, USA*
Hannah Caldwell *Copenhagen, Denmark*
Havovi Chichger *Cambridge, UK*
Silvia Conde *Lisbon, Portugal*
Joseph Costello *Portsmouth, UK*
Jennifer Davies *Cardiff, UK*
Judy Delp *Tallahassee, FL, USA*
Aimee Drane *Cardiff, UK*
Esther Dupont-Versteegden *Lexington, KY, USA*
Andrea Fellet *Buenos Aires, Argentina*
Carrie Ferguson *Torrance, CA, USA*
Igor Fernandes *Niteroi, Brazil*
Ryan Hoiland *Kelowna, Canada*
Seth Holwerda *Kansas City, KS, USA*
Anna Hudson *Adelaide, Australia*
Matthew Kay *Washington, DC, USA*
James Lang *Ames, Iowa, USA*
Jacqueline Limberg *Columbia, MO, USA*
Fiona McBryde *Auckland, NZ*
Carmen De Miguel *Birmingham, AL, USA*
Niels Olesen *Copenhagen, Denmark*
Leah Reznikov *Gainesville, FL, USA*
Kleiton Silva *Camden, NJ, USA*
Sarah Short *Iowa City, IA, USA*
Nathaniel Szewczyk *Nottingham, UK*
Can Ozan Tan *Boston, MA, USA*
Mike Tymko *Vancouver, Canada*
Kai Yang *Guangzhou, China*
Song Yao *Melbourne, Australia*
Gensheng Zhang *Hangzhou, China*

**FUTURE LEADERS**
Edward Ashworth *Basel, Switzerland*
Sophie Broome *Melbourne, Australia*
Callum Brownstein *Newcastle, UK*
Daniel Craighead *Minneapolis, MN, USA*
Robert Lakin *Toronto, Canada*
Jedd Pratt *Manchester, UK*
Fabio Sarto *Padua, Italy*
Hollie Speer *Canberra, Australia*

**CONSULTANT EDITORS**
Richard Burden *Bisham, UK*
Nic Green *Bedfordshire, UK*
Julie Greeves *Salisbury, UK*
Denis Noble *Oxford, UK*
Nanduri Prabhakar *Chicago, IL, USA*

**PUBLISHER**
Alex Stewart (astewart@physoc.org)

**DEPUTY MANAGING EDITOR**
Hollie Evans (hevans@physoc.org)

**PEER REVIEW MANAGER**
Diana Jones (ephjournal@physoc.org)

**PEER REVIEW COORDINATOR**
Paroma Guha (ephjournal@physoc.org)

**EDITORIAL OFFICE**
The Physiological Society
Hodgkin Huxley House
30 Farringdon Lane, Clerkenwell
London EC1R 3AW

**PRODUCTION OFFICE**
K. Nandini (EPH@wiley.com)

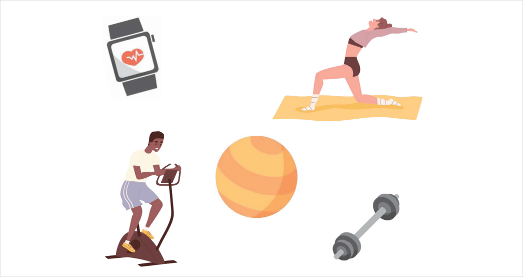

**Cover Illustration**
A representation of the Exercise as Medicine special issue. This special issue was organised Professor Ronan Berg (University of Copenhagen), Dr Colleen Deane (University of Southampton), Dr Jill Barnes (University of Wisconsin-Madison) and Professor Harry Rossiter (The Lundquist Institute for Biomedical Innovation at Harbor-UCLA Medical Center).

**Open Access and Copyright**
All articles published by Experimental Physiology [submitted after 18 October 2022] are fully open access: immediately freely available to read, download, and share. All articles [accepted from 18 October 2022] are published under the terms of a Creative Commons license.
Copyright on any research article published by Experimental Physiology is retained by the author(s).
Further information about open access licenses and copyright can be found at: https://authorservices.wiley.com/author-resources/Journal-Authors/licensing/licensing-info-faqs.html#4. Information on the licenses used by Experimental Physiology can be found at: https://physoc.onlinelibrary.wiley.com/journal/1469445X.

For submission instructions and all other information visit: https://physoc.onlinelibrary.wiley.com/journal/1469445X

The Physiological Society and The Physiological Society logo are trade marks belonging to The Physiological Society and are registered in the UK and in the EU Community, respectively.

*Experimental Physiology* publishes high quality, original, physiological research papers that give novel insights into fundamental homeostatic and adaptive responses in health and papers that further our knowledge of pathophysiological mechanisms in diseases.

Experimental Physiology
Open Access
Integration and Translation

### INFORMATION AND GUIDANCE FOR AUTHORS

**Full version online at http://ep.msubmit.net/cgi-bin/main.plex?form_type=display_requirements**

### ORIGIN

*The Quarterly Journal of Experimental Physiology*, founded in 1908 by Sir Edward Sharpey-Schafer, Professor of Physiology, University of Edinburgh, became a publication of The Physiological Society in 1981. The title was changed to *Experimental Physiology* in 1990.

### SCOPE

*Experimental Physiology* publishes research papers that report novel insights into homeostatic and adaptive responses in health, as well as those that further our understanding of pathophysiological mechanisms in disease. We encourage papers that embrace the journal's orientation of translation and integration, including studies of the adaptive responses to exercise, acute and chronic environmental stressors, growth and aging, and diseases where integrative homeostatic mechanisms play a key role in the response to and evolution of the disease process. Examples of such diseases include hypertension, heart failure, hypoxic lung disease, endocrine and neurological disorders. We are also keen to publish research that has a translational aspect or clinical application. Comparative physiology work that can be applied to aid the understanding human physiology is also encouraged.

Manuscripts that report the use of bioinformatic, genomic, molecular, proteomic and cellular techniques to provide novel insights into integrative physiological and pathophysiological mechanisms are welcomed.

We are also interested in methodological papers reporting important new developments in physiological techniques that could lead to significant new insights.

Papers which observe a significant new physiological behaviour or phenomenon which has the potential to alter the understanding of that field, but which have not yet identified the mechanisms may also be considered. Such papers should demonstrate the experimental examination and exclusion of a number of plausible mechanisms for the important novel phenomenon.

### Article Types

In addition to original research, *Experimental Physiology* consider the following article types for publication:

*Case studies, Short Communication, Connections, Reviews, Full Reviews, Hot Topic Reviews Myths and Methodologies and Symposium Reports.*

Review articles are normally by invitation but proposals may be submitted for consideration.

Commentary articles in the form of Viewpoints and Letters are also published.

For full guidelines on all published article see full guidelines.

C O P E COMMITTEE ON PUBLICATION ETHICS

This journal is a member of and subscribes to the principles of the Committee on Publication Ethics

### ETHICS OF EXPERIMENTATION

**Animal Experiments** - see full journal policy at https://physoc.onlinelibrary.wiley.com/hub/animal-experiments

Experimental Physiology endorses the ARRIVE Guidelines for reporting *in vivo* experiments: http://onlinelibrary.wiley.com/doi/10.1113/expphysiol.2010.053793/full

Authors of papers submitted to Experimental Physiology must adhere to the journals' principles and standards for reporting animal experiments.

Authors should read these guidelines to ensure that their research and experiments adhere to our exacting standards.

Euthanasia/humane killing: Animals must be killed using methods approved for that species, stage of development and size. The standards required by the journals are those of the UK/European Union legislation, set out in in ASPA Schedule 1 in the UK and in Annex IV in the European Directive 2010/63/EU. Methods not on Schedule 1 or Annex IV could be permissible but even where the method used conforms with national guidelines, the onus is on the author to provide scientific evidence to demonstrate that it is at least as humane as those on Schedule 1 and Annex IV.

Our guidelines have been outlined in the Editorial 'Principles and standards for reporting animal experiments in Experimental Physiology'.

**Confirmation of compliance**

Authors must state in the Methods section that the investigators understand the ethical principles under which the journal operates and that their work complies with the animal ethics checklist as outlined in the Editorial.

**Human Experiments** - See full journal policy at https://physoc.onlinelibrary.wiley.com/hub/human-experiments

The Editorial Boards are concerned that all work published in The Journal of Physiology and Experimental Physiology should have been conducted according to the highest standards of safety and ethics. Legislation and accepted practice concerning human experimentation vary from country to country and consequently it is difficult to define absolute requirements. However, work with human subjects should conform to the standards set by the Declaration of Helsinki (last modified in 2013), the Medical Research Council's online guidance on the use of human tissue, and the guidelines set out below. The objectives must be to ensure that all risks are minimized and that subjects are not injured and do not feel they have been abused as a result of participating in the study. Any definition of abuse will include excessive or unexpected pain or discomfort experienced during the experiment. Note, in the case of experiments involving minors, any risks must have been considered absent or minimal, and evidence must be presented that the experiments were performed with the understanding and consent of the legal guardian.

All manuscripts must contain statements indicating that (i) informed consent has been obtained, preferably in writing, (ii) studies conformed to the standards set by the Declaration of Helsinki or the version that was in place at the time of the experiments, NOTE: If the research study was registered (clause 35 of the Declaration of Helsinki) the registration database should be indicated, else the lack of registration should be noted as an exception (e.g. The study conformed to the standards set by the Declaration of Helsinki, except for registration in a database.); and (iii) the procedures have been approved by the local ethics committee.

### DISCLAIMER

The Publisher, the Physiological Society and Editors cannot be held responsible for any errors in or any consequences arising from the use of information contained in this journal. The views and opinions expressed do not necessarily reflect those of the Publisher, the Physiological Society or Editors, neither does the publication of advertisements constitute any endorsement by the Publisher, the Physiological Society, Editors, or Authors of the products advertised.

Wiley Open Access articles posted to repositories or websites are without warranty from Wiley of any kind, either express or implied, including, but not limited to, warranties of merchantability, fitness for a particular purpose, or non-infringement. To the fullest extent permitted by law Wiley disclaims all liability for any loss or damage arising out of, or in connection with, the use of or inability to use the content.

### Experimental Physiology

Vol 111.6

### Contents of volume 111.6

1 June 2026

### EDITORIAL

2737 *Experimental Physiology* Special Issue: Exercise as Medicine
*Ronan M. G Berg, Colleen S Deane, Jill N Barnes and Harry B Rossiter*
2744 Muscle fibre type shift in COPD: Adaptive, maladaptive or a bit of both?
*Jacob Peter Hartmann, Hannah G. Caldwell and Ulrik Winning Iepsen*
2748 Contemporary concepts in 'exercise as medicine' and related fields
*Daniel H. Craighead, Casper Simonsen, Grit Elster Legaard, Cody G. Durrer, Helga Ellingsgaard, Andre Nyberg, Jill N. Barnes, Colleen S. Deane, Harry B. Rossiter, Damian M. Bailey, Bente Klarlund Pedersen and Ronan M. G. Berg*

### VIEWPOINT

2757 Favouring respiration over locomotion in chronic obstructive pulmonary disease
*Kiana M. Schulze and David C. Poole*

### SHORT COMMUNICATION

2759 Alveolar–capillary reserve in COPD assessed by the pulmonary diffusing capacity response to an upright-to-supine postural change
*Iben Elmerdahl Rasmussen, Stine Buus Nymand, Jacob P. Hartmann, Rie Skovly Thomsen, Helene Louise Hartmeyer, Amalie B. Andersen, Milan Mohammad, Birgitte Hanel, Jann Mortensen, Ulrik Winning Iepsen and Ronan M. G. Berg*

### REGISTERED REPORT PROTOCOL

2777 Clinical significance of exercise-induced hypoalgesia in individuals with temporomandibular disorders and neck pain: A clinical trial protocol
*Luiz Felipe Tavares, Ana Izabela Sobral de Oliveira-Souza, Vladimir Aron, Ana Beatriz Oliveira, Henrik Bjarke Vaegter and Susan Armijo-Olivo*

### REVIEW ARTICLE

2787 Immune system benefits of pulmonary rehabilitation in chronic obstructive pulmonary disease
*Asghar Abbasi, David Wang, William W. Stringer, Richard Casaburi and Harry B. Rossiter*
2805 Exercise training to preserve vitality capacity in ageing
*Raymond Jones, Taylor L. Taylor, Robert T. Mankowski, Fitzgerald Dodds, Michael Hankes, Joanna Hobson, Yi Lin, Keith Saffold, Silvienne C. Sint Jago, McKenna A. Tharpe, Emily L. Zumbro, Anna Thalacker-Mercer and Thomas W. Buford*
2813 The therapeutic role of exercise training during menopause for reducing vascular disease
*Conan L. H. Shing, Bert Bond, Kerrie L. Moreau, Jeff S. Coombes and Jenna L. Taylor*
2828 Physical activity and joint health: Implications for knee osteoarthritis disease pathophysiology and mechanics
*Karl Morgan, Joshua Carter, Dario Cazzola and Jean-Philippe Walhin*
2840 Vascular health and exercise in females throughout the lifespan: Exploring puberty, pregnancy and menopause
*Kathleen B. Miller, M. Erin Moir and Brandon G. Fico*
2860 Locomotor muscle dysfunction and rehabilitative exercise training in fibrotic interstitial lung disease: Where are we at and where could we go?
*Sarah Thivent, J. Alberto Neder, Anne-Catherine Bernard, Marie Coudurier, Michel Guinot, Frédéric Hérengt, Samuel Verges and Mathieu Marillier*
2880 Respiratory muscle endurance training among patients with chronic diseases: A systematic review of available evidence
*Hélène Laurent, Frédéric Costes, Ruddy Richard and Marc Filaire*
2895 Cross-education of lower limb muscle strength following resistance exercise training in males and females: A systematic review and meta-analysis
*Abdulmajeed Altheyab, Helal Alqurashi, Timothy J. England, Bethan E. Phillips and Mathew Piasecki*
2918 The effectiveness of aerobic exercise on pain and disability in individuals with neck pain: A systematic review and meta-analysis
*Ana Izabela Sobral de Oliveira-Souza, Marie Kempe, Sofia Grimmelsmann, Luiz Felipe Tavares, Ester Moreira De Castro-Carletti, Angela Viegas Andrade, Liz Dennett, Harry Von Piekartz, Jorge Fuentes Contreras and Susan Armijo-Olivo*

### RESEARCH ARTICLE

#### CARDIOVASCULAR CONTROL

2945 Effects of high-intensity interval training on cardiac function in hypertensive and normotensive men: Effects of antihypertensive treatment
*Mads Fischer, Jon Egelund, Matteo Fiorenza, Thomas S. Ehlers, Michael Nyberg, Jesper J. Linde, Lasse Gliemann, Thomas P. Gunnarsson and Jens Bangsbo*

#### ENDOCRINOLOGY AND METABOLISM

2956 One minute of light-intensity stair-stepping decreases postprandial glycaemia in the evening in non-diabetic adults: A randomized controlled trial
*Austin Morales, William Wong, Jeff Moore and Jochen Kressler*
2963 Assessing the exercise-related kinetics of circulating cell-free DNA, circulating tumour DNA, DNase I activity and cytokines in patients with solid tumours: A pilot study
*Elmo W. I. Neuberger, Alexandra Brahmer, Tobias Ehlert, Suzan Botzenhardt, Alfonso De Falco, Birgit Enders, Patricia S. Hähnel, Achim Heintz, Carl C. Schimanski, Thomas Kindler and Perikles Simon*

### ENVIRONMENTAL AND EXERCISE

2975 Impact of a 12-week high-intensity interval training intervention on cardiac structure and function after COVID-19 at 12-month follow-up
*Iben Elmerdahl Rasmussen, Mathilde Løk, Cody Garett Durrer, Anna Agnes Lytzen, Frederik Foged, Vera Graungaard Schelde, Josephine Bjørn Budde, Rasmus Syberg Rasmussen, Emma Fredskild Høvighoff, Villads Rasmussen, Mark Lyngbæk, Simon Jønck, Rikke Krogh-Madsen, Birgitte Lindegaard, Peter Godsk Jørgensen, Lars Køber, Niels Vejlstrup, Bente Klarlund Pedersen, Mathias Ried-Larsen, Morten Asp Vonsild Lund, Ronan M. G. Berg and Regitse Højgaard Christensen*

2987 Test–retest reliability of Doppler ultrasound-based leg blood flow assessments during exercise in patients with chronic obstructive pulmonary disease
*Milan Mohammad, Jacob P. Hartmann, Amalie B. Andersen, Helene L. Hartmeyer, Ulrik W. Iepsen and Ronan M. G. Berg*

3000 Impact of exercise training on tumour-infiltrating T cells in human prostate cancer: A secondary analysis of a randomized controlled trial (PRO-TEST)
*Simon Nørskov Thomsen, Emil Wriedt, Marianne Gardar Stærk, Sissal Sigmundsdottir Djurhuus, Birgitte Grønkær Toft, Sabrina Wielsøe, Andreas Røder, Thomas Hasselager, Peter Busch Østergren, Henrik Jakobsen, Klaus Brasso, Jesper Frank Christensen, Louise Lang Lehrskov and Casper Simonsen*

3010 The modified Borg cycle strength test (mBCST): Feasibility and physiological response in people with COPD and healthy older adults
*Jana De Brandt, Johan Jakobsson, Mattias Hedlund, Thomas Sandström and André Nyberg*

3026 Exercise-induced miR-126 expression improves vascular health in prediabetes: A randomized controlled trial
*Elif Yildirim Ayaz, Sadrettin Pençe, Sibel Kuraş, Emine Şeyma Denli Yalvaç, Banu Mesci, Özden Ezgi Üner, Fatoş Nimet Kaya, Berna Dincer, Ferruh Kemal Işman and Aytekin Oğuz*

### MUSCLE

3039 Myocellular adaptations to short-term weighted wheel-running exercise are largely conserved during C26-tumour induction in male and female mice
*Stavroula Tsitkanou, Pieter Koopmans, Calvin Peterson, Ana Regina Cabrera, Ruqaiza Muhyudin, Francielly Morena, Sabin Khadgi, Eleanor R. Schrems, Tyrone A. Washington, Kevin A. Murach and Nicholas P. Greene*

### NEUROSCIENCE

3055 Feasibility of superimposed supine cycling and lower body negative pressure as an effective means of prolonging exercise tolerance in individuals experiencing persisting post-concussive symptoms: Preliminary results
*Raelyn Javra, Joel S. Burma, Nathan E. Johnson and Jonathan D. Smirl*

3069 The acute effect of two exercise modalities on neurocognitive responses in postmenopausal women: A randomized controlled trial
*Morgane Le Bourvellec, Nathalie Delpech, Laurent Bosquet, Geoffroy Boucard and Carina Enea*

### VASCULAR

3082 Resistance exercise breaks during prolonged sitting augment the blood flow response to a subsequent oral glucose load in sedentary adults
*Emily M. Rogers, Nile F. Banks, Emma R. Trachta, Morgan S. Wolf, Alexander C. Berry, Anna E. Stanhewicz, Lucas J. Carr, Bethany Barone Gibbs and Nathaniel D. M. Jenkins*

### LETTER TO THE EDITOR

3099 Letter to the editor on exercise and immune competence: using pre-clinical findings to inform the design of clinical studies
*Amit Hagar*

Editor's Pick

A representation of the Exercise as Medicine special issue. This special issue was organised Professor Ronan Berg (University of Copenhagen), Dr Colleen Deane (University of Southampton), Dr Jill Barnes (University of Wisconsin-Madison) and Professor Harry Rossiter (The Lundquist Institute for Biomedical Innovation at Harbor-UCLA Medical Center).
